# Effect of *Origanum vulgare* Essential Oil Supplementation on the Advanced Parameters of Mobility and on the Integrity of Human Sperm DNA

**DOI:** 10.1155/2020/1230274

**Published:** 2020-05-13

**Authors:** Modou M. Mbaye, Bouchra El Khalfi, Saida Ouzamode, Brahim Saadani, Noureddine Louanjli, Abdelaziz Soukri

**Affiliations:** ^1^Laboratory of Physiopathology, Genetics Molecular and Biotechnology (PGMB), Faculty of Sciences Ain Chock, Research Center Health and Biotechnology, University Hassan II of Casablanca, Morocco; ^2^In Vitro Fertilization Center IRIFIV, IRIS Clinic, Casablanca, Morocco; ^3^Laboratory of Medical Analyses, Reproductive Biology, Labomac, Casablanca, Morocco

## Abstract

The reduced sperm mobility is one of the most important causes of male infertility. Several reports have indicated that the treatment of subnormal sperm samples with certain agents prior to artificial insemination significantly improves the fertilizing potential of sperm. We have among others some stimulants such as pentoxifylline, relaxin, prostaglandin E, and diltiazem. In our precedent work, we had tested the effect of supplementation with three essential oils, namely, sage (*Salvia officinalis*), oregano (*Origanum vulgare*), and eucalyptus (*Eucalyptus globulus*), on sperm cell mobility and vitality. Oregano oil had shown interesting biological properties by giving the best values of progressive mobility and vitality. In this study, we aim to verify the effect of oregano oil supplementation on the advanced parameters of mobility and on the integrity of the sperm DNA of 25 male infertile volunteers. Our results showed that oregano oil over an incubation period of 5 to 10 min of exposure significantly improves the advanced parameters of mobility, namely, curvilinear velocity (VCL), linear velocity (VSL), the mean velocity of the path (VAP), and the amplitude of the displacement (ALH). The effect of the increase in the VCL decreased the linearity (LIN), the mean line (STR), and the mean wobble (WOB). Oregano oil at 5 min had no significant effect on the DNA fragmentation index (DFI) and sperm decondensation index (SDI). However, at 10 min, it had a significant effect on both DFI and SDI. The analysis of our results showed that this plant oil rich in terpenoids and phenolic antioxidants could be a quite good in vitro additive with high potential for the world of medically assisted reproduction.

## 1. Introduction

Asthenozoospermia characterized by a sperm cell mobility deficit is one of the major causes of male infertility [1]. This is due to a decrease in energy levels that can be caused by a failure of the main pathway of ATP hydrolysis, damage to sperm cell structures (flagella), or infections [2]. To overcome this energy deficiency, experts in reproductive biology propose many solutions, often of a chemical nature, thus encountering toxicity problems.

Finding natural biomolecules capable of improving sperm mobility and vitality in vitro is a promising, attractive, and very decisive path for a significant improvement in the low results of in vitro fertilization, which are stagnating at less than 20%.

Our previous work had shown that the in vitro supplementation of the essential oil of oregano during 5 and 10 min of incubation at 37°C under 5% of CO_2_ significantly improved the mobility and the vitality of the spermatozoa [3].

Advanced mobility parameters, especially the different speeds, are positively correlated with sperm/oocyte interaction [4].

The rate of sperm DNA fragmentation and the level of sperm decondensation have a significant impact on embryo development and survival.

In order to show that oregano could be a potential beneficial additive for the preparation of sperm in vitro for the world of medically assisted reproduction, we considered necessary to study the effect of oregano oil supplementation on the advanced parameters of sperm cell mobility, namely, curvilinear velocity (VCL), linear velocity (VSL), mean path velocity (VAP), range of motion (ALH), linearity (LIN), mean straight line (STR), and mean wobble (WOB), as well as its impact on sperm DNA integrity through evaluation of the DNA fragmentation index (DFI) and the sperm decondensation index (SDI).

## 2. Materials and Methods

### 2.1. Chemical Products

All chemicals used in this study were obtained from Sigma-Aldrich (St. Louis, MO, USA).

### 2.2. Plant Material

Essential oil (EO) was obtained directly from the leaves of *Origanum vulgare L*. plants (Fliyou) collected at the Fez region (central Morocco). The plant material was randomly collected, washed, and dried in a well-ventilated place at room temperature for ten days before being used according to the method of Sabir et al. [5]. The sample was then isolated and stored for extraction.

### 2.3. Essential Oil

The essential oil was obtained by hydrodistillation 3.5 h using the Clevenger apparatus according to the method of Sabir et al. [5]. The oil was extracted from the distillate with hexane and dehydrated by passing through anhydrous sodium sulphate. After filtration, the solvent was removed by reduced pressure distillation in a rotary evaporator at 35°C, and the pure oil was stored in an amber bottle stored in the refrigerator (4°C), until use [5]. The nonlethal concentrations of the essential oil were determined by the dilution technique in series according to the method of Mar et al. [6].

### 2.4. Sample Collection

This study was carried out at the Laboratory of Medical Analysis and Reproductive Biology, “Labomac,” Casablanca, Morocco. We have established a study group of 25 samples of men diagnosed with Asthenozoospermia (≥20 × concentrations × 106 ml; progressive motility is <32%). Informed consent was obtained from all patients included before using their sperm in this study. Then, samples were collected after 3 to 4 days of abstinence in sterile and labelled containers [3]. For liquefaction, the samples were stored at 37°C under 5% CO_2_ until use. We checked at a time interval of 10 min until the liquefaction was done. The microscopic analysis was performed in accordance with World Health Organization (WHO) standards and guidelines [3].

### 2.5. Semen Treatment

**Figure 1:**
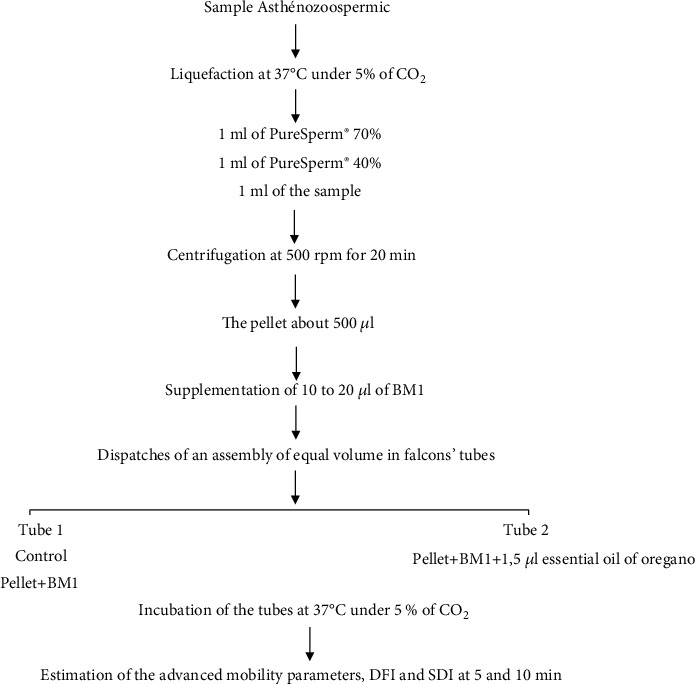
Sample processing method.

After one hour of sperm production, a routine sperm analysis was performed to determine sperm count, motility, and vitality. To make it, a sample of 20 *μ*l of sperm was deposited in a Makler room. The sperm pretreatment was performed using the density gradient optimization technique. Thus, 1 ml of PureSperm® 70%, 1 ml of PureSperm® 45%, and 1 ml of semen sample were added, respectively, in a 10 ml Falcon tube and centrifuged at 500 rpm for 20 min. The pellet (approx. 0.5 ml) was fortified with 10 to 20 *μ*l of BM1, then divided into 2 equal aliquots in 10 ml Falcon tubes; the first tube containing the pellet and BM1 was incubated as a control; the second was also incubated with 1.5 *μ*l of oregano oil at a final concentration of [10^−3^]. Incubations were performed at 37°C under 5% CO_2_ (Figure 1) [3].

### 2.6. Mobility Analysis

The effect of oregano oil on advanced parameters of human sperm cell mobility has been evaluated at different incubation periods: 0, 5, and 10 min at 37°C under 5% CO_2_. The protocol consists of depositing an aliquot of 20 *μ*l of the mixture (sperm/oil, control) in a preheated Makler chamber and analyzing by CASA (Hamilton-Thorne Semen Computer-Assisted Analysis version 10 HTM IVOS Analyzer (Hamilton-Thorne Biosciences, Beverly, MA, USA)). The parameters used for the analysis are as follows: number of images, 8; frame rate, 20 Hz; layer thickness 10 *μ*l; temperature, 37°C; minimum contrast, 6; minimum size, 6; small size grid, 0.4; large size grid, 2.0; low intensity grid, 2.5; and high intensity grid, 4.3 [1]. Eight representative fields containing at least 100 mobile sperm cells were examined. Throughout the analysis, an integrated heated stage maintained the sample temperature at 37°C [1]. The parameters recorded for each sample were percentage viability, percentage progressive motility, and movement characteristics such as curvilinear velocity (VCL), linear velocity (VSL), mean path velocity (VAP), lateral displacement amplitude (ALH), mean linearity (LIN: VSL/VCL), mean straight line (STR: VSL/VAP), and mean wobble (WOB: VAP/VCL) [1].

### 2.7. Vitality Analysis

The assessment of sperm viability was performed with 2% eosin staining [3]. To do this, semen samples were mixed with an equal volume of eosin solution. A smear on a glass slide was made from the mixture and was allowed to air dry. Immediately after drying, each slide was examined under an optical microscope. The test is performed immediately after contact between the sperm cells and different concentrations of EO (*t* = 0 min), at exposure times ranging from 5 to 10 min, and the preparation was incubated at 37°C under 5% CO_2_ [3].

### 2.8. Measurement of the DNA Fragmentation Index by TUNEL Test

The integrity of the sperm DNA was assessed by the TUNEL test using a commercial kit (Roche Diagnostics, Lewes, United Kingdom) according to the manufacturer's recommendations. The principle of the TUNEL technique is to use an enzyme, the Terminal Deoxynucleotidyl Transferase (TdT), capable of adding nucleotides to the 3′-OH ends of free DNA.

**Figure 2:**
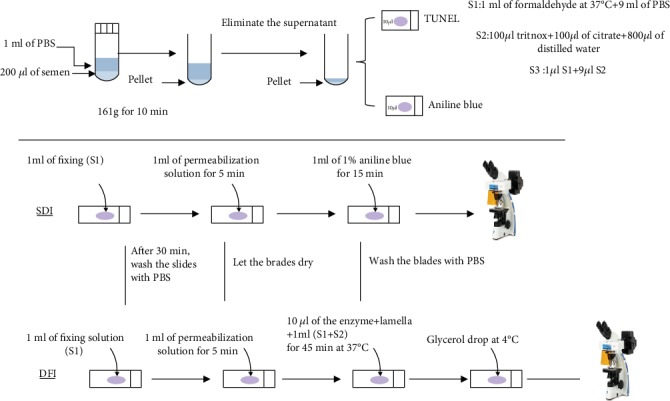
Processing method used for evaluating the sperm decondensation index (SDI) and the DNA fragmentation index (DFI).

The sperm sample was washed twice in phosphate-buffered saline (PBS, Sigma-Aldrich, Gillingham, UK) and adjusted to a concentration of 2 × 10^7^ cells/ml in PBS. The cell suspension was then fixed in PBS containing 2% formaldehyde (Sigma-Aldrich) for 60 min at room temperature. Following a double wash with PBS, the sample is centrifuged at 1200 rpm. This step is repeated twice. Slides prepared were immediately analyzed using a fluorescence microscope (Nikon Eclipse 80i) equipped with appropriate filters. The images were captured using a CCD camera and XytoGen software (Excilone, version 3.8.46, France) (Figure 2) [7].

### 2.9. Measurement of the Index of Sperm Decondensation to Aniline Blue

The slides of the prepared sperm samples were rinsed twice with distilled water and then stained with 5% aniline blue bath pH 3.5 for five min. They are then quickly rinsed with distilled water and then dehydrated in alcohol baths (70, 90, and 100°, one minute each). The reading is done under white light at magnification ×1000 and immersion (Figure 2).

A total of 500 spermatozoa were counted on the slides made from the whole sperm. Positive for aniline blue (a poorly condensed chromatin spermatozoon) is any sperm for which the blue color occupies more than 50% of the surface of the head [7].

### 2.10. Statistical Test

The data obtained during our experiments were the subject of a statistical study. The results of the oregano oil supplementation on the characteristic parameters of mobility and nuclear quality were obtained by the Student *t*-test (*t*-test). All the graphs and histograms represented in this article have been created with the software GraphPad Prism 7.

## 3. Results

### 3.1. Effects of *Origanum vulgare* Oil on Mobility and Vitality

**Figure 3:**
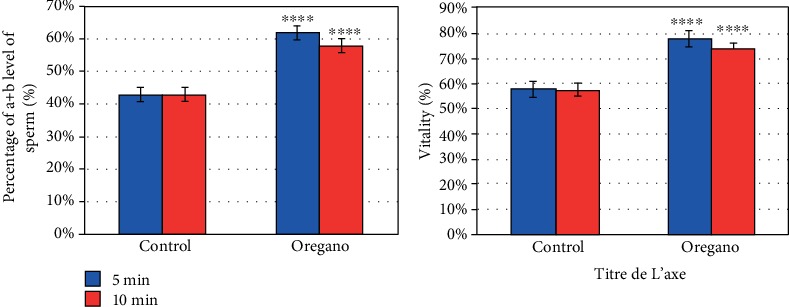
In vitro effect of oregano oil on the proportion of mobile sperm. The dilution of EO was done in a 0.2% (*w*/*v*) sterile agar solution. The percentage motility and vitality of each aliquot was measured after the addition of the EO and 5 min and 10 min treatment. Oregano oil could significantly increase the number of progressive mobile sperm cells and viability over a period of 5 to 10 min (^∗∗∗∗^*P* < 0.001; *n* = 25).

In vitro supplementation of oregano essential oil (*Origanum vulgare*) has significantly improved the percentage of progressive mobility as well as the vitality of human sperm cells (Figure 3) (*P* < 0.001).

### 3.2. Advice Effects of *Origanum vulgare* on Advanced Mobility Parameters

**Figure 4:**
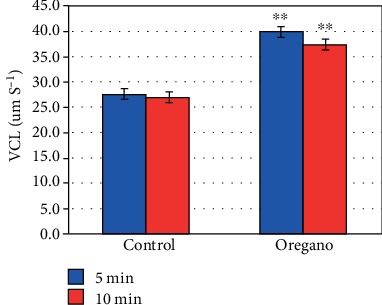
In vitro effects of oregano oil on the curvilinear velocity (VCL). The dilution of the EO was performed in 0.2% (*w*/*v*) sterile agar solution. The percentage of VCL of each aliquot was measured after the addition of the EO and 5 min and 10 min treatment. Oregano oil significantly improves the VCL over a period of 5 to 10 min (^∗∗^*P* < 0.01; *n* = 25).

**Figure 5:**
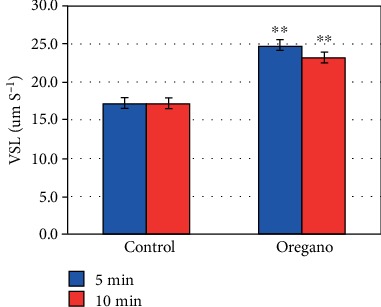
In vitro effect of oregano oil on the linear velocity (VSL). The dilution of EO was done in a 0.2% (*w*/*v*) sterile agar solution. The percentage of VSL of each aliquot was measured after the addition of the EO and 5 min and 10 min treatment. Oregano oil could significantly improve the VSL over a period of 5 to 10 min (^∗∗^*P* < 0.01; *n* = 25).

**Figure 6:**
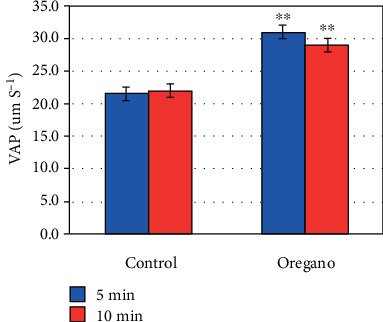
In vitro effect of oregano oil on the mean velocity of the path (VAP). The dilution of EO was done in a 0.2% (*w*/*v*) sterile agar solution. The percentage of VAP of each aliquot was measured after the addition of the EO and 5 min and 10 min treatment. Oregano oil could significantly improve the VAP over a period of 5 to 10 min (^∗∗^*P* < 0.01; *n* = 25).

**Figure 7:**
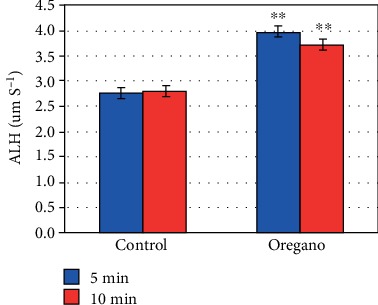
In vitro effect of oregano oil on the amplitude of displacement (ALH). The dilution of EO was done in a 0.2% (*w*/*v*) sterile agar solution. The percentage of ALH of each aliquot was measured after the addition of the EO and 5 min and 10 min treatment. Oregano oil could significantly improve ALH over a period of 5 to 10 min (^∗∗^*P* < 0.01; *n* = 25).

In vitro supplementation of oregano essential oil (*Origanum vulgare*) has shown a significant stimulating effect on advanced mobility parameters curvilinear velocity (VCL) (*P* < 0.01) (Figure 4), linear velocity (VSL) (*P* < 0.01) (Figure 5), mean path velocity (VAP) (*P* < 0.01) (Figure 6), and amplitude of displacement (ALH) (*P* < 0.01) (Figure 7).

**Figure 8:**
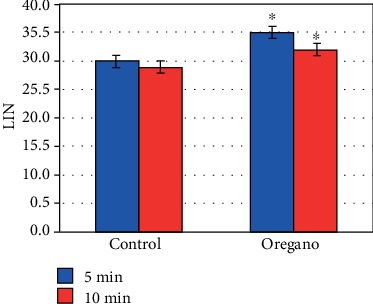
In vitro effect of oregano on the linearity (LIN). The dilution of EO was done in a 0.2% (*w*/*v*) sterile agar solution. The percentage of LIN of each aliquot was measured after the addition of the EO and 5 min and 10 min treatment. Oregano oil could significantly improve LIN over a period of 5 to 10 min (^∗^*P* < 0.05; *n* = 25).

**Figure 9:**
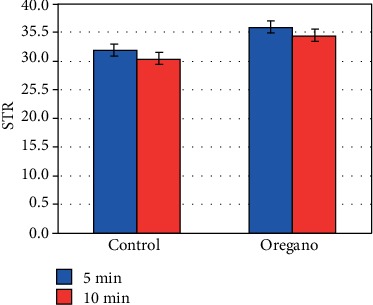
In vitro effect of oregano oil on the mean line (STR). The dilution of EO was done in a 0.2% (*w*/*v*) sterile agar solution. The percentage of STR of each aliquot was measured after the addition of the EO and 5 min and 10 min treatment. The effect of such an increase in VCL has resulted in a substantial reduction in STR, LIN, and WOB.

**Figure 10:**
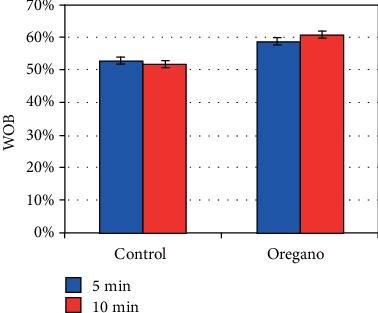
In vitro effect of oregano oil on the mean wobble (WOB). The dilution of EO was done in a 0.2% (*w*/*v*) sterile agar solution. The percentage of WOB of each aliquot was measured after the addition of the EO and 5 min and 10 min treatment (*P* = 0.52; *n* = 25).

The effect of the increased VCL decreased very substantially the linearity (LIN) (*P* < 0.05) (Figure 8), the mean line (STR) (Figure 9), and the mean wobble (WOB) (Figure 10).

### 3.3. Effect of *Origanum vulgare* on DNA Fragmentation Index

**Figure 11:**
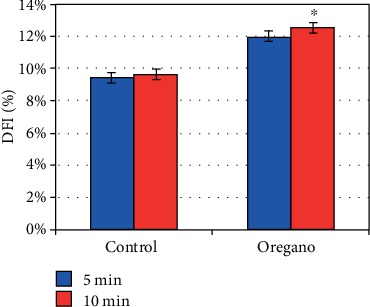
In vitro effect of oregano oil on DNA fragmentation index. DFI was measured after the addition of the EO and 5 min and 10 min treatment. The dilution of EO was done in a 0.2% (*w*/*v*) sterile agar solution. The percentage of DFI of each aliquot was measured immediately after the addition of the EO (*t* = 5 min) (*P* = 0.53; *n* = 25) and 10 minutes later (*t* = 10 min) (^∗^*P* < 0.05; *n* = 25).

Supplementation of oregano in Asthenozoospermic sperm after 5 and 10 min of incubation at 37°C under 5% CO_2_ does not alter the level of sperm with DNA fragmentation. Because the rate of sperm with DNA fragmentation is as high in the fortified sample as in the control, difference is not significant (*P* = 0.53) at 5 min. However, on the other hand, we noted a significant difference at 10 min (*P* < 0.05) (Figure 11).

### 3.4. Effect of *Origanum vulgare* on Sperm Decondensation Index

**Figure 12:**
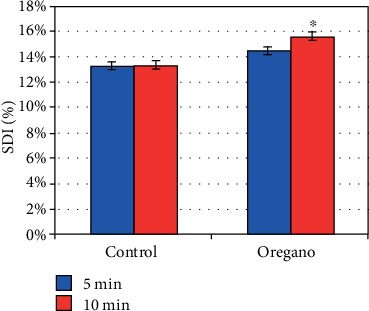
In vitro effect of oregano oil on the sperm decondensation index. SDI was measured immediately after the addition of the EO and 5 min and 10 min treatment. The dilution of EO was done in a 0.2% (*w*/*v*) sterile agar solution. The percentage of SDI of each aliquot was measured after 5 min (*P* = 0.48; *n* = 25) and 10 min (^∗^*P* < 0.05; *n* = 25).

The level of spermatozoa with a condensation defect of DNA after 5 to 10 min of incubation at 37°C under 5% of CO_2_ shows that the in vitro supplementation of oregano oil in human sperm has no deleterious effect on the normal condensation of sperm chromatin since mean values of supplemented and control samples do not show any significant difference (*P* = 0.48) at 5 min. However, we noted a significant difference at 10 min (*P* < 0.05) (Figure 12).

## 4. Discussion

Sperm transfer in the procedures of in vitro fertilization, intrauterine insemination, and intrafallopian gamete is done with washed sperm [1]. Finding methods to optimize sperm for all these procedures is utmost important for professionals in reproductive biology [8].

Oregano essential oil could be included among the many plants reported in the literature with improving effects on human sperm mobility [9].

In our previous work, it has been shown that oregano at 5 min exposure at 37°C under 5% CO_2_ significantly improves the progressive mobility and vitality of human sperm cells (*P* < 0.001) (Figure 3) [3].

Our experiments with oregano oil supplementation on advanced mobility parameters have shown that 5 and 10 min EO exposure at 37°C under 5% CO_2_ significantly improves curvilinear velocity (VCL) (Figure 4), linear velocity (VSL) (Figure 5), mean path velocity (VAP) (Figure 6), and displacement amplitude (ALH) (Figure 7) of sperm cells.

On the other hand, sperm cell linearity (LIN) (Figure 8) as well as the average line (STR) (Figure 9) and the average wobble (WOB) (Figure 10) decreased substantially due to the increase in the VCL and VAP. Our results are in good agreement with those of Barlow and Liu groups [1, 10]. Our ALH values are also in agreement with the work of Liu et al., Aitken and Clarkson, and Bongo et al., by showing that ALH is higher in sperm cells of fertile men than those of infertile men; they show also a positive correlation of the effectiveness of penetration into the cervical mucus of sperm with increased ALH [1, 11, 12].

Several studies have shown that oregano essential oil contains phenolic compounds and flavonoids, in particular, rosmarinic acid, quercetin, kaempferol, apigenin, rutin, and oregano which can cleanse free radicals such as the superoxide anion and hydroxyl [13, 14]. Thus, the improvement in mobility, the advanced parameters of mobility, and the vitality of spermatozoa by the essential oil of oregano can be explained by its antioxidant activity against lipid peroxidation [14]. Moreover, Daghigh Kia et al. have shown that the peroxidation of lipids in sperm damages the structure of the lipid matrix in the membranes of sperm and that it is linked to a rapid loss of intracellular ATP which can lead to axonemal damage and reduced sperm viability [15]. It is in this same endeavor that Aitken determined that the peroxide radical (H_2_O_2_) could be distributed across the membranes of the spermatozoa and thus inhibit the activity of key enzymes such as glucose-6-phosphate dehydrogenase (enzyme which plays a major role in ATP production and sperm motility) [16].

Our ALH values agree well with the work of Liu et al., Aitken and Clarkson, and Bongo et al., by showing that ALH is higher in sperm of fertile men and also show a positive correlation of the effectiveness of penetration into the cervical mucus of sperm with an increase in ALH [1, 11, 12].

The evaluation of the effect of oregano oil supplementation on DNA integrity at 5 min incubation at 37°C under 5% CO_2_ did not yield a significant difference for both DFI (Figure 11) and SDI (Figure 12) values. However, after 10 min of incubation, a significant difference was noted for both DFI (*P* < 0.05) and SDI (*P* < 0.05) with respect to the controls (Figures 11 and 12). The absence of a convention on the threshold values of the DFI and the SDI makes it difficult to interpret them. We can say that oregano essential oil does not impact the index of DNA fragmentation and sperm decondensation index because we have recorded lower values than the positive threshold value for DFI (4%) and for SDI (15%) [17]. Sperm abnormalities can cause not only embryonic development problems [4] but also spontaneous miscarriages [1]. The low DFI and SDI values can result from various factors in addition to the supplementation of oregano oil because even in the control, we noted low DFI and SDI values. Moreover, numerous studies have shown that alteration of sperm DNA can result from various phenomena such as apoptosis, oxidative stress, production of endogenous Reactive Oxygen Species (ROS), and spontaneous and/or exogenous alterations (ionizing radiation, toxic components of the environment), which act on DNA elements by producing a very wide range of damage such as deletions or breaks in free nitrogenous bases [18].

The results of oregano oil supplementation on the advanced parameters of mobility and DNA quality of the sperm show that *Origanum vulgare* essential oil could be a promising alternative for better male infertility management especially in patients with Asthenozoospermia.

## 5. Conclusion

This study highlights the improving effects of in vitro supplementation with oregano essential oil on advanced sperm mobility parameters and its impact on sperm DNA quality. The results allowed us to observe an improvement in the advanced parameters of mobility, DFI and SDI, after 5 min of incubation. Based on the results, we can conclude that oregano oil could be a safe therapeutic alternative for the management of motility dysfunction in Asthenozoospermic patients.

## Data Availability

The data used to support the findings of this study are available from the corresponding author upon request.
